# Service delivery strategies for perinatal mental health in sub-Saharan Africa: A scoping review

**DOI:** 10.4102/hsag.v31i0.3222

**Published:** 2026-04-30

**Authors:** Manoko I. Lediga, Mutshidzi A. Mulondo, Joyce M. Tsoka-Gwegweni

**Affiliations:** 1 Division of Public Health, Faculty of Health Sciences, University of the Free State, Bloemfontein, South Africa

**Keywords:** mental health services, perinatal mental health conditions, perinatal women, strategies, sub-Saharan Africa

## Abstract

**Background:**

Mental health conditions, such as depression and anxiety, affect many women during the perinatal period. These conditions are particularly prevalent in low- and middle-income countries and low-resource settings, where access to quality mental health services is limited. This highlights the urgent need for effective strategies to identify, support and empower women experiencing perinatal mental health difficulties.

**Aim:**

This scoping review synthesised existing evidence on strategies to improve access to perinatal mental health services in sub-Saharan Africa (SSA).

**Method:**

A scoping review was conducted using databases including EBSCOhost, Scopus, PubMed and Web of Science. Studies published between 2015 and 2025 in English were included. Reporting followed the Preferred Reporting Items for Systematic Reviews and Meta-Analyses for Scoping Reviews checklist.

**Results:**

The initial search identified 735 records, of which only four met the inclusion criteria. These studies employed varied approaches, enriching the synthesis by capturing diverse perspectives and methodological insights. This variation strengthened the comprehensiveness and credibility of the findings.

**Conclusion:**

Sustainable improvements in perinatal mental health services in SSA require investment in workforce development, culturally adapted interventions and supportive policies.

**Contribution:**

This review contributes to the growing body of knowledge on perinatal mental health in SSA and offers guidance for future strategies aimed at prioritising mental health services for perinatal women.

## Introduction

The experience of pregnancy, childbirth and transitioning into motherhood is a unique period for women (Gusak, Kendall & Nizalova 2023). While this period is often recognised as a positive life event, some women might experience perinatal mental health conditions (MHCs). Perinatal mental health refers to the mental and emotional health of parents during pregnancy and up to 2 years after a baby is born (Fisher 2023) and includes MHCs such as depression, anxiety, stress and psychosis (Rajak et al. 2023). Perinatal MHCs may encompass pre-existing mental health issues that intensify or deteriorate during pregnancy and the postpartum period. However, they are largely undiagnosed at the primary care level as a result of a range of systemic and contextual challenges, such as mental health training among primary health care providers, stigma surrounding MHCs (Phungula, Mkhize & Mokoena 2024), and because primary health care centres operate without access to mental health specialists or structured support (World Health Organization [WHO] 2022). Globally, approximately 10% of women in high-income countries (HICs) and more than 25% in low- and middle-income countries (LMICs) are affected by perinatal MHCs in the perinatal period (Fisher et al. 2012; Van Heyningen et al. 2019). Studies by Prom et al. (2022) and Ng’oma et al. (2020) highlight that women living in resource-limited environments are particularly vulnerable to perinatal MHCs. This heightened risk is linked to factors such as poverty, exposure to gender-based violence, adverse birth outcomes, and in some cases, food insecurity (Abrahams et al. 2018). Prevention and early intervention benefits for perinatal MHCs have been recognised globally (McGorry & Mei 2018). Goal three of the Sustainable Development Goals aims to ensure healthy lives and promote well-being for all, at all ages. Further targeting ‘to reduce by one-third premature mortality from non-communicable diseases through prevention and treatment, and to promote mental health and well-being’ (United Nations 2023:12). Furthermore, the WHO (2022) developed a guide for clinical managers, district and primary health facilities and nongovernmental organisations that provide maternal and child health services to integration of mental health services in maternal and child health services. McNab et al. (2020) proposed that governments take responsibility for women’s mental health needs by not only developing specific national policies but also implementing them. A situational analysis conducted in five LMICs, Ethiopia, India, Nepal, South Africa and Uganda show a limited capacity of health systems regarding feasible detection and treatment strategies (Baron et al. 2016), further highlighting the need for mental health services. Studies mapping out gaps and pathways for perinatal mental healthcare exist; however, most of these studies were conducted in HICs (Horáková et al. 2024; Reinsperger & Paul 2022). Studies conducted in sub-Saharan Africa (SSA) mainly highlight the need for increased investment and innovation in this area (Bauer et al. 2022; Nakidde, Kizito & Mugisha 2023; Nwoke et al. 2023). While other studies focus mainly on perinatal depression with limited focus on other MHCs (Bitew et al. 2020; Nakku et al. 2021). This scoping review, therefore, synthesises existing evidence on strategies designed to improve access to perinatal mental health services in SSA.

## Methods

### Eligibility criteria

This scoping review collected and analysed peer-reviewed full-text empirical research articles written in English between 2015 and 2025. Included was primary research that was published (journal articles, chapters). Details of the criteria are indicated in Table 1.

**TABLE 1:** Inclusion and exclusion criteria.

Study characteristics	Included	Excluded
Location	Research conducted in the sub-Saharan African countries	Studies conducted outside sub-Saharan Africa
Publication status	All publications, regardless of research design, sample size and methods used, were included	Unpublished Master’s and PhD dissertations and theses; rapid, scoping and systematic reviews
Publication date	2015–2025	Before 2015
Language	English	Non-English
Outcome	Studies focusing on at least two or more common perinatal MHCs	Studies focusing on one common perinatal MHCs (e.g. depression only; anxiety only)
	Including either pregnant women or women in the postnatal period, or both	-
Intervention	Studies focusing on all women in the perinatal period	Studies focusing on a single group of perinatal women (e.g. adolescents/teenagers; women living with human immunodeficiency virus; women experiencing gender-based violence)

MHC, mental health condition.

### Information sources and search strategy

The University of the Free State’s (UFS) online library was utilised to access and search electronic databases. This increased the likelihood of finding research conducted in SSA. The following databases were selected with the assistance of an expert librarian to maximise the likelihood of finding research conducted in SSA, including PubMed, Scopus, Web of Science, EBSCOhost and Google Scholar. Additionally, a hand search was performed, along with a review of the reference lists of included articles, to identify any additional studies missed in the database search.

Keywords used were a combination of Boolean operators ‘OR’ & ‘AND’. Searched words included:

*‘perinatal mental health OR maternal mental health’, AND ‘perinatal mental health services OR maternal mental health services’, AND ‘sub-Saharan Africa OR specific country’ [e.g. South Africa, Egypt, Mali] AND ‘strategies OR interventions’*.

This approach facilitated the retrieval of sources that specifically addressed the relevant subjects. While there are 49 countries in SSA, the extracted studies represented 28 countries, as studies from these countries were the only ones available within the search parameters. It was found that literature from Francophone and Lusophone countries was underrepresented; this may be because of language barriers. As a result, the majority of studies extracted were from the following countries: South Africa, Kenya, Nigeria, Ethiopia, Tanzania, Zimbabwe, Zambia, Malawi, Ghana and Liberia.

### Study selection

**FIGURE 1:**
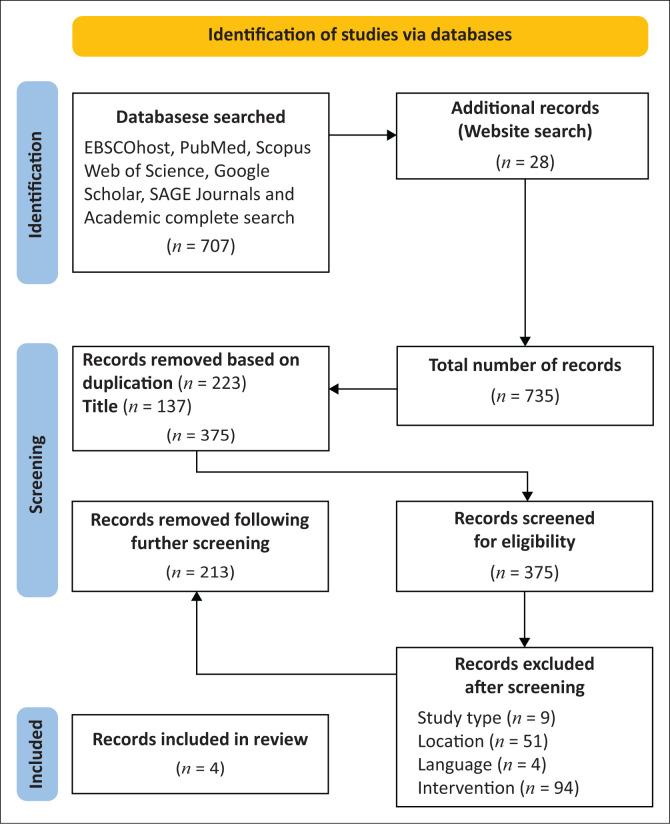
Preferred Reporting Items for Systematic Reviews and Meta-Analyses for Scoping Reviews diagram.

Following a comprehensive literature search, all studies were reviewed to identify those meeting the eligibility criteria. Initially, 735 studies were. After removing duplicates, 512 studies remained for title and abstract screening, during which 137 were excluded based on their titles and/or abstracts. Subsequently, 375 full-text articles were assessed, with 371 excluded for not meeting the inclusion criteria. These were studies that focused on one perinatal MHC and/or on a specific group of women (adolescents/teenagers and/or perinatal women living with HIV and AIDS or diabetes). Ultimately, only four studies fulfilled all eligibility requirements. The four studies included in the synthesis used different approaches, which enriched the synthesis by capturing diverse perspectives and methodological insights. This variation strengthened the comprehensiveness and credibility of the findings. The selection process was documented using a Preferred Reporting Items for Systematic Reviews and Meta-Analyses for Scoping Reviews (PRISMA-ScR) flow diagram (Figure 1) to ensure transparency and replicability.

### Data extraction

Data extraction was conducted by ML, MM and with assistance from a UFS librarian. Relevant data from the selected studies were extracted and organised into a standardised data extraction form. This form included fields for the author(s), publication year, study approach and design, sample size, setting (geographical location and healthcare context) and key findings related to service delivery strategies as shown in Table 2.

**TABLE 2:** Summary of results.

Author	Study design	Sample size	Setting	Strategy	Results	Recommendations
Abrahams et al. (2019)	Mixed-method study (Quantitative analysis and cognitive interviewing)	66 women (first antenatal visit)	Midwife Obstetric Unit, Hanover Park, Cape Town, South Africa	4-item screening tool	There is a need to develop culturally validating screening tools for common perinatal MHCs.	The authors emphasise the need for increased investment in mental health infrastructure, particularly in maternal care settings, and advocate for the integration of mental health services into routine perinatal care, the importance of culturally sensitive interventions, the value of community-based approaches and the training of healthcare providers to better identify and manage perinatal MHCs.
Boisits et al. (2021)	A mixed-method formative study	20 health workers and 37 pregnant women	Across four Midwife Obstetric Units, Cape Town, South Africa	Task-shared, low-intensity psychological counselling intervention	The study informed common counselling elements (problem-solving, psychoeducation, basic counselling skills). The findings highlighted that mental health literacy was low. Many women did not recognise symptoms as mental conditions, often describing then ‘in’ behavioural or physical terms.	Mental health literacy among pregnant women was low. Use of task sharing via CHWs to deliver short, structured psychological interventions for perinatal anxiety and depression within existing public health infrastructure. Incorporate key therapeutic components such as problem-solving, behavioural activation, psychoeducation, and basic counselling skills. Use local idioms/metaphors to increase acceptability.
Honikman et al. (2025)	Case study / descriptive evaluation of a complex intervention	N/A	Low-resource, high-volume public health maternity facilities, Cape Town, South Africa	A collaborative, stepped-care model: Routine screening for perinatal MHCs (depression, anxiety); Task-sharing with non-specialist providers; Counselling and referral services	The strategies demonstrated the feasibility of embedding mental health care into routine maternity services. Overall, there was an increased identification of women with perinatal mental health concerns. As a result, a total of 414 sessions were conducted with mental health counsellors for engagement, assessment and triage. Psychotherapy and social support were offered to 392 women, of whom 348 accepted.	To scale up integrated mental health services in primary care, train non-specialist providers, and use stepped care to optimise resources.
Van Heyningen et al. (2019)	A cross-sectional diagnostic tool development study	376 pregnant women	Primary-level obstetric clinic, Cape Town, South Africa	A 9-item ultra-short screening tool	The tool demonstrated strong psychometric validity for detecting antenatal depression, anxiety and suicidal ideation.	Implementation of screening tools in low-resource settings can improve early detection and intervention for common perinatal MHCs.

Note: Please see full reference list of this article: Lediga, M.I., Mulondo, M.A. & Tsoka-Gwegweni, J.M., 2026, ‘Service delivery strategies for perinatal mental health in sub-Saharan Africa: A scoping review’, *Health SA Gesondheid* 31(0), a3222. https://doi.org/10.4102/hsag.v31i0.3222 for more information.

MHC, mental health condition; CHW, Community health workers.

### Ethical considerations

Ethical approval for the scoping review was not necessary; however, it was obtained as a waiver under a larger study for the ongoing PhD research.

## Results

### Description of the relevant studies

Table 2 describes studies and interventions in terms of inclusion and exclusion criteria. Included studies encompass different research designs, including two mixed-method designs, one case study, and one cross-sectional diagnostic tool development study. All these four studies included women of all ages; however, they largely focused on pregnant women, excluding women in the postnatal period. The studies also describe different types of strategies, which include screening, stepped care models (screening, counselling, and referral services integrated into routine antenatal care) and task sharing. All the studies were conducted in Cape Town, South Africa.

Table 2 describes studies in terms of inclusion and exclusion criteria, location, publication size and date, language, outcome, and intervention.

## Discussion

Addressing perinatal mental health through evidence-based strategies is essential for improving the well-being of women. Effective strategies, including screening, stepped care models (screening, counselling, and referral services integrated into routine antenatal care) and task sharing, can lead to early identification and management of symptoms, thereby reducing negative outcomes (Abrahams et al. 2019; Boisits et al. 2021; Honikman et al. 2025; Van Heyningen et al. 2019).

### Strategies related to mental health services for perinatal women

#### Screening

Routine screening for mental illness is the most common strategy; however, in most countries, only pregnant women are screened with little focus on the postpartum period (Schneider et al. 2018). In their study, Honikman et al. (2025) highlighted the impacts of screening, where women were routinely screened for mood disorders during their antenatal visits. Women who screened positive were referred to on-site counsellors, who additionally served as case managers. Counselling sessions were scheduled by appointment, and support was available for up to 1 year after childbirth. Each woman who received counselling was also contacted through a routine follow-up phone call 6 weeks after delivery (Honikman et al. 2025). This strategy was proven to be effective and impactful, as about 90% of women who visited this facility between July 2008 and the end of June 2011. Screening instruments have also proven to be effective when used in Mali and Uganda (Lasater et al. 2020; Nakku et al. 2021). In their study, Abrahams et al. (2019) demonstrated that the Edinburgh Postnatal Depression Scale (EPDS) remains a useful and practical tool for detecting symptoms of depression in perinatal women. The EPDS was designed for populations with low literacy or different cultural conceptions of mental health. Therefore, the EPDS was found to be inclusive, particularly in settings where literacy levels are low and cultural understandings of mental health are different. This aligns with research findings that highlight the importance of contextual adaptation of screening tools in LMICs (Baron et al. 2016). Both studies, however, underscore that screening alone is insufficient unless followed by appropriate referral systems and treatment pathways (Abrahams et al. 2019; Van Heyningen et al. 2016). The authors also noted that screening alone is insufficient. This echoes the broader consensus that screening should be linked to appropriate mental health services, especially in overburdened and under-resourced public health systems (Docrat et al. 2019; Honikman et al. 2025). While the EPDS is a validated tool, its implementation has remained inconsistent within South African public healthcare facilities, highlighting significant implementation gaps. In addition, scholars such as Abrahams et al. (2019) and Van Heyningen-Pienaar (2021) found that healthcare providers reported having limited training and expressed low confidence in administering screening tools, underscoring the urgent need to prioritise capacity-building initiatives in this area.

#### Task sharing

Scaling up primary mental healthcare is recommended in the South African mental health policy (Phungula, Robertson & Mall 2024). Task sharing mitigates the critical shortage of mental health professionals in LMICs, expanding access to evidence-based perinatal mental healthcare by empowering trained non-specialist providers to deliver structured interventions (Patel et al. 2018). Task sharing has proven to be an effective and scalable approach to addressing service gaps (Boisits et al. 2021). As demonstrated by Boisits et al. (2021), integrating task sharing into primary healthcare settings offers an effective approach to managing mild to moderate perinatal depression and anxiety, especially in contexts with limited resources. Earlier formative work in Khayelitsha, Cape Town, also found that task-sharing was both feasible and acceptable (Schneider et al. 2016).

These strategies collectively provide a practical and scalable framework to improve the reach, quality and impact of perinatal mental health care, particularly in low-resource settings. Implementing these strategies effectively requires multisectoral collaboration, political will, and sustained investment. While task sharing has emerged as a strong strategy, particularly in the context of perinatal care, several gaps may undermine its effectiveness. For example, Honikman et al. (2025) and Van Heyningen et al. (2019) found that healthcare providers often receive limited training and supervision, which affects the quality and consistency of support they deliver. Additionally, in low-resource settings where staff shortages are evident, implementing and following task sharing models can be challenging (Baron et al. 2016).

### Stepped care models: Counselling and referral services

In their study, Honikman et al. (2025) demonstrated how integrating counselling and referring patients can improve access and outcomes for women experiencing psychological distress. Counselling includes psychoeducation, emotional support sessions and problem-solving. Within healthcare environments, counselling may be delivered by trained health workers or non-specialist providers such as community health workers and mentor mothers. Similarly, Devkota et al. (2017) found that counselling significantly enhances positive outcomes during pregnancy by improving women’s knowledge, shaping more supportive attitudes, and promoting safer health practices. Referral services complement counselling by ensuring that women with mental health needs are connected to appropriate services. More recently, a study conducted by Honikman et al. (2025) highlighted that stepped care models enhanced service delivery and approachability to maternal mental health needs in primary maternity care. Even so, gaps remain in the consistency of these services. For example, Van Heyningen et al. (2019) found that referral pathways were unclear, there is a limited availability of mental health professionals, especially within the public sector, and training and supervision inadequacies persist. As a result, these strategies may collectively provide a practical and scalable framework to improve the reach, quality and impact of perinatal mental health care, particularly in low-resource settings. Implementing them effectively requires multisectoral collaboration, political will and sustained investment.

## Recommendations

- As supported by research, mental health screening should be routinely integrated into maternal and child health services. This integration can ensure early detection and timely management of MHCs during the perinatal period.
- Task sharing should be expanded by training non-specialist health workers, such as nurses, midwives and lay counsellors, to deliver evidence-based interventions (Boisits et al. 2021; Schneider et al. 2016). Task sharing has the potential to address the critical shortage of mental health professionals and may increase service coverage in underserved communities (Boisits et al. 2021).
- Strategies should be community-led, culturally sensitive and co-developed with stakeholders to ensure acceptability, relevance and sustainability. Involving trusted community members increases engagement and helps reduce stigma around mental health.
- There should be clear pathways, developed by health and systems to ensure the identification, referral, treatment and follow-up of perinatal MHCs for what? For this to happen, collaboration across levels of care is essential to ensure continuity and comprehensiveness.
- Lastly, given that all four studies included in this study were conducted in one city, future research should aim at including participants from other regions and/or countries as a way of exploring whether findings are consistent across different cultural and policy environments.

## Conclusion

Improving perinatal mental health care in SSA and other low-resource settings requires a multifaceted and context-specific approach. Evidence shows that strategies such as task sharing, routine screening and counselling and referral services are feasible and effective in addressing service gaps for perinatal women (Abrahams et al. 2019; Boisits et al. 2021; Honikman et al. 2025; Van Heyningen et al. 2016). Integrated into primary healthcare systems, these strategies may facilitate the early detection and management of perinatal MHCs. However, to ensure sustainability and impact, there is a need for investments in capacity-building for lay providers, culturally adapted tools and interventions, and robust monitoring systems. Ultimately, prioritising perinatal MHCs is not only crucial for the well-being of mothers but also for child development and broader community health. With sustained commitment and evidence-based implementation, progress in this area can contribute significantly to achieving global health equity and improving outcomes in maternal mental health.
